# Black Soldier Fly (*Hermetia Illucens*) as Dietary Source for Laying Quails: Live Performance, and Egg Physico-Chemical Quality, Sensory Profile and Storage Stability

**DOI:** 10.3390/ani9030115

**Published:** 2019-03-25

**Authors:** Antonella Dalle Zotte, Yazavinder Singh, Joris Michiels, Marco Cullere

**Affiliations:** 1 Department of Animal Medicine, Production and Health, University of Padova, Agripolis, Viale dell’Università 16, 35020 Legnaro, Padova, Italy; yazavinder.singh@phd.unipd.it (Y.S.); marco.cullere@unipd.it (M.C.); 2 Department of Animal Sciences and Aquatic Ecology, Faculty of Bioscience Engineering, Ghent University, Coupure Links 653, 9000 Gent, Belgium; joris.michiels@ugent.be

**Keywords:** black soldier fly, insect meal, alternative protein, animal feeding, quail, egg quality, nutritional composition, fatty acid profile, oxidation

## Abstract

**Simple Summary:**

The search for alternative and sustainable protein sources in poultry production is an issue of major importance due to the increasing demand of products of animal origin and, consequently, of feedstuffs to feed food-producing animals. The latter is exacerbating the overexploitation of natural resources, thus increasing the environmental impact of animal farming, as well as the prices of raw materials. In this sense, insects have been indicated as one of the possible alternatives to solve this problem, thus research efforts to test the feasibility of a practical application to the poultry sector are required. Therefore, the present study tested different inclusion levels of a defatted black soldier fly larvae protein meal in the diets for laying quails as an alternative ingredient to the soybean meal and oil. Results of the present study showed that the insect meal can technically be applied to laying quails’ feed formulations, providing optimal performance and health status. Egg nutritional quality and sensory profile too did not worsen when quails were fed with the insect meal supplementation. Further research efforts should be performed to improve the healthiness of the lipid fraction of the tested insect larvae and, in turn, the lipid quality of the eggs obtained from insect-fed quails.

**Abstract:**

Insects are promising candidates as alternative sustainable sources of protein for poultry species. The present research studied the effect of a dietary inclusion of a defatted black soldier fly (BSF) larvae meal as an alternative protein source in the diets of laying quails, on productive performance, egg physicochemical quality, fatty acid profile, sensory traits and storage stability. A total of 225 laying quails were divided into 3 dietary groups (5 replicates/each). A conventional soybean meal-based diet was formulated (Control group), and two other diets were formulated including either 10% (BSF10) or 15% (BSF15) defatted BSF larvae meal. Laying quails showed satisfactory productive performance throughout the trial. BSF10 and BSF15 eggs had the highest shape index (*p* < 0.01), shell weight and percentage (*p* < 0.001) and the most intense yolk color (*p* < 0.001). Defatted BSF larvae meal increased the eggs’ saturated fatty acid content (*p* < 0.001) to the detriment of the polyunsaturated fraction (*p* < 0.001). Overall the eggs’ sensory profile was not affected by the dietary treatment, but BSF15 eggs had a higher feed off-flavor *vs* Control group (*p* < 0.05). At day 28 of storage, oxidative stability was higher in BSF10 vs. Control eggs (*p* < 0.01). Defatted BSF larvae meal can be considered a possible alternative ingredient to soybean meal in laying quail diets, up to the 15% inclusion level.

## 1. Introduction

In the last years, insects have started being considered a part of the possible solution to directly alleviate food insecurity, but also to improve the sustainability of the livestock production. It has been demonstrated that if insects are farmed exploiting low value food processing by-products or high-impacting waste streams, their use in animal feeding can be environmentally more beneficial than traditional sources of proteins [1]. Insects are a natural dietary source of nutrients for many animal species, including free-range poultry and wild birds [2]. Despite this, insect-based feeds in the European Union are currently allowed only for aquaculture, whereas further research and thus legislative efforts to allow their utilization in poultry species is still required. One of the most promising and widely investigated insect species in this sense is the black soldier fly (*Hermetia illucens*). This *Diptera* can be reared on a wide range of decomposing organic waste that are not suitable for human nutrition, like by products from food processing and organic waste [3]. In this way the black soldier fly (BSF) can reduce the potential environmental load of waste streams, converting them into high valuable proteins (41–44% of the total dry matter), which could be used in feed formulations [4]. The protein derived from this efficient insect is also rich in essential amino acids, and it showed an overall superior amino acid profile compared to soybean meal [5]. Furthermore, a recent study found that despite its high methionine: cysteine ratio (61:39), a complete replacement of soybean meal with BSF larva meal in broiler diets provided satisfactory outcomes without the need to modulate the optimal methionine: cysteine ratio [6]. But BSF larvae are not only a source of protein; in fact, they have a high fat content (up to 35%), and they are rich in minerals, among which Ca (5–8% dry matter -DM-) and P (0.6% to 1.5% DM) [4].

Because of all these positive features, together with being an insect species suitable for mass production, different research studies have been performed to evaluate the potential of including BSF in poultry diets. BSF meal was found to be an excellent source of energy, digestible amino acids, for broilers chickens [7], broiler quails [2,8], and Barbary partridge [9], ensuring optimal health status and proving satisfactory growth performance and overall meat quality. Among widely consumed animal products, eggs are a low-cost source of high-quality protein, lipids, vitamins and minerals, thus being suitable for any kind of consumer [10]. Because of this, their production is rapidly increasing, and it is expected to reach the 89 million ton by 2030 [11]. For this reason, the BSF larvae have also started being studied as a possible alternative ingredient for laying hens. Recent research studied layers’ performance, intestinal morphometry, enzymatic and microbial activity, egg quality and sensory traits [11,12,13,14,15,16], and encouraging results were provided.

Despite this, the influence of BSF larvae meal in laying quails’ diets is not clear yet. Quail production has been increasing in the last years, thanks to a series of positive intrinsic characteristics of this avian species: It provides high quality meat and eggs with fast return on investment, thanks to the early sexual maturity, rapid growth, short generation interval, high laying rate and limited feed and space requirements per bird [17,18]. 

Based on these premises, the present research aimed at providing a holistic overview of the potential application of defatted BSF larvae meal in the diet for laying quails. The effects of two different defatted BSF larvae meal inclusion levels (10% and 15% in the diet) on the productive performance of the animals and the physicochemical quality, sensory attributes and storage stability of the eggs, were evaluated. 

## 2. Materials and Methods

### 2.1. Experimental Design

The experiment was conducted in a laying quails farm in the Veneto region of Italy (“La Colombara” Società Agricola, Castelnovo di Isola Vicentina, VI, USA), after the approval by the veterinary authority, and in accordance with the article 2, DL 4 March 2014, No. 26 of the Official Journal of the Italian Republic (http://www.gazzettaufficiale.it/eli/id/2014/03/14/14G00036/sg), implementing the EC Directive 86/60963/2010 EU regarding the protection of animals used for experimental and other scientific purposes.

The experimental design consisted of three treatments of different experimental diets: A control diet (Control), which was formulated referring to the common layer diet used in the farm, and two diets supplemented with 10% and 15% of defatted black soldier fly (BSF) larvae meal (BSF10 and BSF15, respectively). Diets were formulated to meet or exceed the minimum requirements for laying Japanese quails [19]; they were in mash form and provided *ad libitum* throughout the experiment. The inclusion levels were chosen based on a previous study on broiler quails [2], and then adjusted to be as much isoenergy and isonitrogenous as possible. The insect meal was purchased from a leading European company specializing in insects as a nutritional source. The Company guarantees hazard analysis and critical control points (HACCP) standards; in addition, the company is certified for Good Manufacturing Process (GMP+). The ingredients of the experimental diets are presented in Table 1.

**Figure 1:**
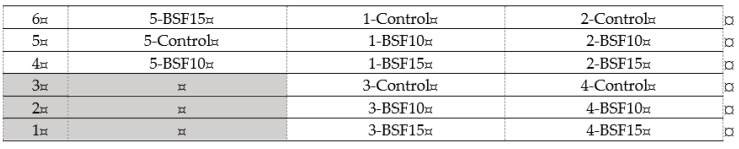
Frontal view of the battery cages used for laying quails: Cage number (replicate; 1 to 5) and treatments (Control, BSF10, BSF15).

Note: the grey colour and empty rectangles means that in those cages no quails were farmed.

Six-month-old laying quails (*Coturnix coturnix japonica*) (n = 225) were individually weighed (initial live weight) and uniformly assigned to the three treatments, providing five replicates of 15 laying quails each. They were kept in battery cages with constant access to water and feed. The adopted photoperiod was 19 hours light: 5 hours dark. Animals were fed with the experimental diets for six weeks. The frontal view of the battery cages is shown in Figure 1.

### 2.2. Productive Performances

Laying quails were individually weighed at the 5^th^ week of dietary treatment, to monitor live weight change along the experiment. Feed intake was recorded weekly on a cage basis. Also, mortality was monitored along the trial. During this period the laid eggs per cage were counted and individually weighed daily; average egg weight was then calculated. Egg production was computed as n laid eggs/n laying quails × 100. Defected eggs (i.e., broken, without solid shell, with unusual shape) were also daily counted and the percentage on the total laid eggs/cage was calculated. The feed conversion ratio was calculated as kg of feed consumed/kg of egg produced. At the 6^th^ week, the eggs were collected in five consecutive days, then marked and analyzed for physicochemical quality, sensory profile and storage stability. A total of n = 210 eggs/treatment were used for physicochemical analysis, n = 60 eggs/treatment for the sensory analysis and n = 16 eggs/treatment for the storage stability.

### 2.3. Egg Physical Analyses

Eggs were transported to the laboratory of the Animal Medicine, Production and Health Department of the University of Padova (Italy) and were individually weighed. Further measurements were: Egg equatorial diameter (mm), and egg height (mm), using a digital caliper (Juwel Schraubtechnik, EB, Werkstraße 14, 57537 Wissen, Altenkirchen, Rheinland Palatinate, Germany) (0–150 mm—Juwel). Such measurements were used to calculate the shape index: equatorial diameter/height × 100. Surface area was calculated as follows: = 3.9782 × (egg weight)^0.7056^ [20]. 

After physical measurements, the eggs were broken, and the eggshell was dried with a paper towel and weighed (±0.1 g), then eggshell thickness (mm) was measured at equatorial level with the digital caliper. The shell percentage was calculated as shell weight/egg weight × 100. The weight of the egg and of the edible portion (calculated as egg weight minus shell weight) were used to obtain the edible portion percentage: Edible portion (g)/egg weight (g) × 100. The pH of the albumen (±0.1) was determined in duplicate, and yolk color was evaluated by comparison with the 15-points Roche Yolk Color Fan (DSM, Wurmisweg 576, CH-4303 Kaiseraugst, Switzerland).

### 2.4. Chemical Analysis of the Defatted BSF Larvae Meal And of the Experimental Diets

The chemical composition of the BSF larvae meal can be found in Table 2, whereas the chemical composition and the fatty acid (FA) profile of the experimental diets are provided in Table 3 and Table 4, respectively. Analyses of the defatted BSF larvae meal and the experimental diets were carried out in duplicate using Association of Official Analytical Communities (AOAC) (*Official methods of analysis*, 17th edition, Association of Official Analytical Chemists, Arlington, VA, United States) [21] methods to determine DM (method no. 934.01), CP (method no. 2001.11) and ash (method no. 967.05). Starch (amyloglucosidase-α-amylase, method no. 996.11) content was analyzed only in the experimental diets. Ether extract (EE) was determined after acid hydrolysis [22]. Gross energy (GE) was measured with an adiabatic bomb calorimeter [23].

The lipid extraction of the diets was performed by Accelerated Solvent Extraction (M-ASE) using petroleum ether as the solvent. The FA profile was determined as described by Cullere et al. [8]. Samples were trans-methylated using a methanolic solution of H_2_SO_4_ (4%), in order to determine fatty acid methyl esters (FAME). A biphasic separation was obtained by adding 0.5 mL of distilled water and 1.5 mL of N-heptane to each sample. FAME were quantified by gas chromatography (Shimadzu GC17A, 1, Nishinokyo-Kuwabara-cho, Nakagyo-ku, Kyoto 604-8511, Japan), equipped with an Omegawax® 250 column (30 m × 0.25 µm × 0.25 µm) (Supelco Inc., Bellefonte, PA, United States) and FID detector. Helium was used as the carrier gas at a constant flow of 0.8 mL/min. The injector and detector temperatures were both 260 °C. Peaks were identified based on commercially available FAME mixtures (37-Component FAME Mix, Supelco Inc., Bellefonte, PA, USA). The results are expressed as % of total detected FAME. 

### 2.5. Chemical Analyses of the Eggs

To guarantee enough material to perform all the scheduled analyses, the content of seven eggs was homogenized to one sample. Twelve samples/per treatment were considered, and they underwent freeze drying. The proximate composition of egg samples was analyzed in accordance with the AOAC [27] methods. The cholesterol content was determined through absolute quantitative analysis using high-performance liquid chromatography (HPLC) following the method described by Casiraghi et al. [28]. The FA profile of the eggs was analyzed following the same method previously described for the experimental diets, with the only exception that it used a binary mixture of solvents (hexane: Isopropanol 3:2). Then, the results expressed as % of total detected FAME and the total lipid content of the samples were used for the quantitative determination (mg/100 g egg) of FA. 

### 2.6. Storage Stability Trial

The storage stability trial was carried out on 16 eggs/treatment. At egg collecting date (n = 8 eggs/treatment) and at day 28 of storage (n = 8 eggs/treatment), albumen pH and yolk thiobarbituric acid-reactive substances (TBARs) were measured. The pH was analyzed with a portable pH meter FG2-Five GoTM (Mettler Toledo, Greifensee, Switzerland) which was calibrated at pH 4.0, 7.0, 9.0 and 12. The extent of egg yolk lipid oxidation (TBARs) was evaluated with a spectrophotometer (Hitachi U-2000; Hitachi, Mannheim, Germany) set at 532 nm, that measured the absorbance of TBARs and a 1,1,3,3-tetraethoxypropane calibration curve [29]. Oxidation products were quantified as malondialdehyde (MDA) equivalents (mg MDA/kg egg yolk).

### 2.7. Sensory Analysis

Eggs were transported to the Sensory Analysis Division of the “Istituto per la Qualità e le Tecnologie Agroalimentari, Laboratorio Analisi Sensoriale (Veneto Agricoltura)”, Thiene, Vicenza, Italy. They were subjected to a descriptive sensory analysis, to detect possible differences among the dietary treatments. The sensory analysis was conducted on 60 eggs/treatment, and two days of analysis were scheduled (30 eggs/treatment/session). Eggs of each session were daily collected in the farm. The eggs were cooked (boiled) for 5 min, cooled by placing them under tap water, then peeled, and the yolk was separated from albumen and served to 10 panelists in random sequence. For every session, each panelist evaluated a total of 9 eggs (3 eggs/treatment). 

Samples were identified by a random three-digit code. Panelists were qualified as experts according to ISO 8586 and had experience with descriptive tests (ISO 13299) on various food matrices. All judges who perform tests with accredited methods undergo training every three years. Panelists underwent two pre-test training sessions of 1 h each to familiarize with the matrices, and select appropriate descriptors, also drawn from the literature. Furthermore, a list of possible off-flavors was also prepared: Off-flavors were chosen according to the expertise of the panel on the tested food product, and to the sensory characteristics of the feed (poultry feed). The eggs used for the training sessions were obtained from quails fed with a conventional diet: They were handled and cooked in the same manner of the samples which were used for the subsequent sensory analysis.

The panel received a list of descriptors to score on numerical and continuous ten cm-long scales from 1 (the lowest score for each descriptor) to 10 (the highest score for each descriptor). The selected descriptors were: Odor intensity, aroma intensity, sweetness, saltiness, solubility and adhesiveness for yolk; odor intensity, aroma intensity, saltiness and consistency for albumen. Furthermore, for each sample, the panel was asked to indicate if and which of the listed tastes and off-flavors they could recognize. The chosen descriptors were: Bitterness, sourness, metallic, poultry, grass, feed, fish, rancid for yolk; bitterness, metallic, poultry, feed for albumen. The evaluation sheet, distribution of samples to the judges and data acquisition were performed using FIZZ software (BIOSYSTEMES FRANCE, St-Ouen l’Aumône, France) installed in ten terminals in the tasting booths of the laboratory. Still water at room temperature and unsalted crackers were available to panelists throughout each sensory session.

### 2.8. Statistics

Performance data and egg physicochemical traits were subjected to two different one-way Analysis of Variance (ANOVA) with experimental diet (Control, BSF10 and BSF15) as fixed effect, following the GLM procedure of the SAS 9.1.3 statistical analysis software for Windows [30]. For performance data, the experimental unit was the cage, whereas for egg physicochemical traits the experimental unit was the sample. A χ^2^ test with Marascuilo [31] procedure was performed on mortality to detect the differences among treatments. A mixed model (PROC MIXED) was used to detect any dietary influence on sensory analysis scores, therefore considering experimental diet and the ten panelists as fixed and random effects, respectively. Least square means were obtained using a Bonferroni test, and the significance was calculated at a 5% confidence level. The χ^2^ test with Marascuilo [31] procedure was also performed on off-flavors characterization to detect the differences among treatments.

## 3. Results

### 3.1. Productive Performances

The results on the productive performances of laying quails fed with increasing levels of defatted BSF larvae meal are shown in Table 5. Overall, the considered productive parameters weren’t affected by the dietary treatments. The average final live weight of the laying quails was 356 ± 4.5 g in the three experimental groups. Considering the 5-week feeding period, the average feed conversion ratio was 3.47 ± 0.1, quails produced an average of 384 ± 7 eggs, weighing 14.4 ± 0.2 g/each, with an average egg production of 74.8 ± 1.2%. The percentage of defected eggs averaged 2.18% and was not affected by the dietary treatment (*p* > 0.05). Mortality was similar in the three dietary groups of laying quails with an average value of 6.22 ± 2.8%.

### 3.2. Egg physical Analyses

Table 6 shows the effects of a dietary inclusion with 10% and 15% defatted BSF larvae meal on eggs’ physical traits. The dietary treatment significantly affected some of the studied traits which were egg shape index (*p* < 0.01), shell weight (*p* < 0.001), shell percentage (*p* < 0.001) and yolk color (*p* < 0.001). 

Independently to the inclusion level, quails fed with defatted BSF larvae meal produced more elongated eggs compared to those of the Control group (76.3 and 76.3 vs. 75.3% for BSF10, BSF15 and Control groups, respectively; *p* < 0.01). 

Defatted BSF larvae meal-fed quails also produced eggs with superior shell weight compared to those of the Control group (1.51 and 1.52 vs. 1.44 g for BSF10, BSF15 and Control groups, respectively; *p* < 0.001), which caused a higher shell percentage compared to the Control group (10.4 and 10.5 vs. 10.1% for BSF10, BSF15 and Control groups, respectively). Also, yolk color increased with the increase in the inclusion level of defatted BSF larvae meal: Values were 4.99 vs. 5.57 vs. 5.81 for Control, BSF10 and BSF15 groups, respectively (*p* < 0.001).

### 3.3. Eggs Chemical Quality

Table 7 shows the effect of a dietary inclusion with 10% and 15% defatted BSF larvae meal on eggs’ proximate composition and cholesterol content. Eggs’ lipid and cholesterol contents were not affected by the dietary treatments. Differently, the protein content of quails’ eggs decreased with increasing the inclusion level of defatted BSF larvae meal: 12.1 vs. 12.1 vs. 11.9 g/100 g egg for Control, BSF10, BSF15 groups, respectively (*p* < 0.05). Conversely, the ash content increased with increasing the inclusion level of defatted BSF larvae meal: 1.11 vs. 1.19 vs. 1.29 g/100 g egg for Control, BSF10, BSF15, respectively (*p* < 0.05). 

### 3.4. Egg Fatty Acid Profile

The FA profile of the quail eggs (% of total FAME) greatly differed depending on the inclusion level of the defatted BSF larvae meal (Table 8). Increasing the incorporation level of defatted BSF larvae meal into laying quail diets, the total saturated fatty acids (SFA) increased (36.1 vs. 37.8 vs. 38.6% of total FAME for Control, BSF10, BSF15, respectively; *p* < 0.001), which was attributable to the increasing proportions of C12:0, C14:0, and C16:0 (*p* < 0.001) FA.

The total monounsaturated fatty acid (MUFA) content followed a similar trend as it progressively augmented with increasing the dietary BSF inclusion level (35.9 vs. 38.6 vs. 40.0% of total FAME for Control, BSF10, BSF15, respectively; *p* < 0.001). This result was determined by the significant increase of C14:1 *n*-9, C16:1 *n*-9, and C18:1 *n*-11 (*p* < 0.001) FA.

For the total polyunsaturated fatty acids (PUFA) an inverse situation was observed as it decreased from the Control to the BSF15 groups (25.5 vs. 20.8 vs. 18.7% of total FAME for Control, BSF10, BSF15, respectively; *p* < 0.001). This was due to a decrease both in the *n*-6 (23.5 vs. 19.2 vs. 17.7% of total FAME for Control, BSF10 and BSF15, respectively; *p* < 0.001) and in the *n*-3 (2.07 vs. 1.65 vs. 1.20% of total FAME for Control, BSF10 and BSF15, respectively; *p* < 0.001) fractions. As the reduction in the *n*-6 PUFA was less intense than what it was observed at for *n*-3 PUFA, the ratio *n*-6/*n*-3 worsened with the inclusion of defatted BSF larvae meal, with no differences between the BSF10 and BSF15 groups. 

Considering singles *n*-6 PUFA, increasing amounts of defatted BSF larvae meal into layers’ diets significantly lowered the C18:2 *n*-6 (*p* < 0.001) and C20:4 *n*-6 (*p* < 0.05) proportions. The reduction of the overall *n*-3 percentage with increasing the dietary incorporation of defatted BSF larvae meal, was due to the reduction of C18:3 *n*-3 (*p* < 0.001) and of C22:6 *n*-3 (*p* < 0.001) FA, whereas the C20:3 *n*-3 followed an opposite trend: 0.16 vs. 0.18 vs. 0.20% of total FAME for Control, BSF10, BSF15, respectively (*p* < 0.05).

As a result of the consistent changes in the FA profile according to the dietary treatment, the health indexes of quails’ eggs were also influenced: The atherogenicity index (AI) and the thrombogenicity index (TI) increased, thus worsened, with increasing the inclusion level of defatted BSF larvae meal (*p* < 0.001). The dietary inclusion of a defatted BSF larvae meal worsened also the HH ratio (*p* < 0.001). As a result of a growing SFA content from the Control to the BSF15 groups, the peroxidability index (PI) linearly decreased, thus making the eggs of the BSF15 quails less susceptible to oxidative phenomena than those of the BSF10 and Control groups (35.1 vs. 40.5 vs. 46.9 for BSF15, BSF10 and Control groups, respectively; *p* < 0.001). 

Table 9 shows the effects of increasing the dietary inclusion with defatted BSF larvae meal (up to 15%) in quails’ diet on the FA profile of their eggs, expressed as mg/100 g egg. As it was previously observed for the FA expressed as a percentage of total FAME, the defatted BSF larvae meal greatly changed the total SFA (*p* < 0.05), MUFA (*p* < 0.05) and PUFA (*p* < 0.001) contents, but mainly the highest inclusion level. Total SFA increased with the dietary incorporation of the defatted BSF larvae meal, but the difference became significant only between the Control and the BSF15 group, whereas BSF10 was intermediate. These changes were mainly due to the C12:0, C14:0, C16:0 and C22:0 (*p* < 0.001) FA. A similar pattern was observed also for the total MUFA, which differed only in the Control compared to the BSF15 groups (1125, 1235, 1288 mg/100 g egg for Control, BSF10, BSF15 groups, respectively; *p* < 0.05). For this change the main FA involved were the C14:1 *n*-9, C16:1 *n*-9, and C18:1 *n*-11 (*p* < 0.001). Differently from the other two main lipid classes, total PUFA content linearly decreased with increasing dietary defatted BSF larvae incorporation levels (800 vs. 664 vs. 601 mg/100 g egg for Control, BSF10, BSF15 groups, respectively; *p* < 0.001). This trend was mainly due to a redction of the C18:2 *n*-6, C18:3 *n*-3 and C22:6 *n*-3 (*p* < 0.001) FA. 

### 3.5. Storage Stability Trial

Results presented in Table 10 show that the albumen pH significantly increased during the storage period (8.71 and 9.44 at days 0 and 28 of storage, average values of the three dietary groups, respectively; *p* < 0.001), but it was not affected by the dietary treatment.

The inclusion of either 10% or 15% defatted BSF larvae meal in the diets for laying quails did not affect the yolk oxidative status at day 0 of storage. Differently, when yolk oxidative status was analyzed again at 28 days of storage, TBARs values showed the lowest value in the BSF10 group and the highest in the Control eggs, with BSF15 being intermediate (1.72 vs. 1.60 vs. 1.66 mg MDA/kg egg for Control, BSF10 and BSF 15 groups, respectively; *p* < 0.01). Day of storage significantly affected only the TBARs value of the BSF10 eggs which were lower at 28 days of storage compared to the value recorded at day 0 (1.60 vs. 1.82 mg MDA/kg egg for BSF10 group ad day 28 and 0 of storage, respectively; *p* < 0.001).

### 3.6. Sensory Analysis

Results of the descriptive sensory analysis performed on eggs obtained from laying quails fed with increasing dietary inclusions of a defatted BSF larvae meal are depicted in Table 11 and Table 12. Overall, the dietary treatment did not affect the considered variables neither for the yolk, nor for the albumen as similar values were recorded for Control, BSF10 and BSF15 eggs. Off-odors and off-flavors perception too remained unaffected by the dietary treatments, with the sole exception of the “feed” off-flavor, which increased from the Control to the BSF15 groups: Control eggs showed a lower value compared to BSF15 ones, with BSF10 eggs showing an intermediate score (5.55 vs. 13.9 vs. 19.4, for Control, BSF10 and BSF15 groups, respectively; *p* < 0.05).

## 4. Discussion

The black soldier fly larva is a nutrient-rich feed source: Values regarding the protein and fat content obtained in the present study for a defatted larva meal are in line with data retrieved from the literature for this type of product [32]. Even if, with age, feed efficiency and laying performance of quails are expected to decline progressively, overall results of laying quails of the present study were satisfactory, considering that they had a feed efficiency of 3.47, which was better than the value of 3.59 recorded in 9-week-old laying Japanese quails [33].

Results of the present study were similar to those of Maurer et al. [15] in Lohmann Selected Leghorn layers fed defatted BSF larva meal (crude protein: 59.0%; crude fat: 11.0%) replacing the 50% or 100% of soybean cake (3-week feeding experiment), as no effect due to the dietary treatment was observed on hens’ live weight, mortality, egg production, feed intake, egg weight. Differently, another study testing a defatted BSF larva meal (crude protein: 61.3%; crude fat: 4.61%) as a complete replacement of soybean meal in the diets for Lohmann Brown Classic laying hens, highlighted a reduction in feed intake which negatively affected final body weight, laying percentage and egg weight [14]. The visual system is well developed in poultry species: It has been reported that they can discriminate feeds with different colorimetric characteristics, that they tend to prefer the feed whose color is similar to the diet fed after hatching, and that colored diets can determine a reduction in feed intake [34]. Despite the biological bases of color preferences in Japanese quail not completely being understood yet, it is accepted that color vision is a predominant aspect in feed choice for them, and that color preferences can be modified by learning and selective breeding [35]. The fact that the defatted BSF larvae meal used in the present trial did not determine a significant change in the final color of the feed, could explain why productive performance of laying quails were not affected, differently from what was observed in the above-mentioned study. Like our findings, no different feed intake related to the use of defatted BSF larva meal in the diet for different strains of laying hens was observed in the experiment by Al-Qazzaz et al. [12] and Mwaniki et al. [16].

Independently to the dietary treatment, the weight of the eggs produced by quails in the present experiment was higher (14.5 g) than values commonly found in literature [18]. Birds of the present study were almost at the end of their productive career, whereas most research studies considered 8–12 weeks-old birds [33]. In quails, egg weight was reported to increase up to 12 weeks of age then remain stable, with different lines, feeding and management strategies determining a certain variability in this sense [17]. 

Ensuring or improving the quality of the eggshell of laying poultry species is of great interest because a good eggshell quality prevents the economic losses due to cracking or damaging phenomena. In this sense, physical parameters such as shell thickness, shell weight and egg shape index play a major role [36,37]. Despite the inclusion of defatted BSF larva meal increasing the egg shape index, results were in line with values reported for this bird species [38] and close to the optimal range 72–76% indicated for hens’ eggs [36]. Results of the present study for which eggs with a higher shape index had also a higher shell weight, agreed with the results of Yilmaz et al. [39], and were a positive correlation between egg shape index and shell weight that was observed.

Feeding increasing levels of defatted BSF larvae meal to layer quails intensified yolk color, which was in line with the observation of Secci et al. [11] on eggs derived from laying hens fed with a 100% dietary substitution of soybean meal with defatted BSF larva meal. On the one hand this result could have been due to the higher maize content of the feed included with the BSF larva meal in both experiments. On the other hand, in the above-mentioned study the increase in yolk redness was justified by the total carotenoid content of the insect meal, which enhanced also the total carotenoid content of the egg yolk. Existing research showed that the BSF larvae can contain 2.00–2.15 mg carotenoids/kg [11,40], but the effect of a dietary supplementation of BSF larva meal in laying birds on the pigment content of the egg is limited to the above-mentioned study by Secci et al. [11], thus highlighting that further efforts to elucidate this aspect are needed, as it could have important market implications. In fact, carotenoids are important pigments that provide attractive color to egg yolk, which is a desirable visual characteristic, and the use of natural sources instead of synthetic ones is certainly more and more appreciated by modern consumers [41]. Furthermore, carotenoids are well known antioxidants thanks to their ability to quench singlet oxygen and trap peroxyl radicals, as well as playing a role in improving the health status of humans though preventing cardiovascular diseases, cancer and other chronic diseases [42].

The proximate composition of quail eggs of the present study was in line with existing data about this avian species [43]. The observed difference in terms of protein content in BSF15 eggs compared to the Control ones was not attributable the protein content of the experimental diets as it was similar, being 194 and 195 g/kg in the two feeds, respectively. Therefore, to explain such finding it was hypothesized a lower protein availability, due to the chitin content of the diets which increased with the defatted BSF larvae meal inclusion level. Chitin, which is a polysaccharide constituting insect exoskeleton, is known to exert a negative effect on nutrient digestion, included the protein fraction which was observed also in the work by Cullere et al. [2] on meat quails fed with 10 and 15% defatted BSF larvae meal. This observation, however, was in contrast with the results of Secci et al. [11], as they observed no effect of a 100% dietary substitution of soybean meal with BSF larva meal in the diets for laying hens on the egg proximate composition.

Results of the present research contrast with the previous cited paper also regarding the ash content of the eggs: In the present study ash augmented with increasing the dietary inclusion level of BSF larva meal, whereas in the above-mentioned research ash content was unaffected by the dietary treatment. A hypothesis to explain the present finding was that the BSF larva meal could have provided a dietary mineral enrichment thanks to the high content in Ca, P, K and Mg [40]. However, Ca and P contents were similar in the three experimental diets and the overall ash content too. Therefore, the reason for such ash content increase remains to be understood. 

The cholesterol content in eggs yolk is species-related: Limiting the comparison to table eggs, quail eggs contain 11.4% more cholesterol (on average 390 mg/100 g whole egg) than dark-shell hen eggs (350 mg/100 g whole egg) [44]. Cholesterol values observed in the present experiment for quail eggs (on average 473 mg/100 g whole egg) were even higher, having 35% more cholesterol than the dark-shell hen eggs of the above-mentioned study. The cholesterol content is primarily regulated endogenously (de novo synthesis) and scarcely dependent on the dietary treatment [45]. Results of the present study agreed with this, as the dietary cholesterol provided to laying quails due to the incorporation of BSF larva meal did not modify the egg cholesterol content. 

However, previous research testing BSF larva meal as a feed ingredient in laying hens (100% substitution of soybean meal), observed a reduction of 11.7% in the cholesterol content of the yolk in hens receiving the experimental diet including the BSF, compared to a Control group fed on soybean meal [11]. Dietary chitin showed to attract negatively charged bile acids and free fatty acids, determining a reduction in the serum cholesterol content, which is the mechanism that should also explain the cholesterol reduction in the yolk [15]. The limited data available on this topic, together with the contrasting results, highlight the need for further research on this aspect.

The nutritional composition of the BSF larvae, including its lipid content, is known to be strongly affected by the rearing substrate [46]; despite this, the FA profile of this insect species is intrinsically characterized by a greatest proportion of SFA, which can account for the 75% of total FA, with C12:0 (lauric), C14:0 (myristic) and C16:0 (palmitic) acids being the main contributors. Moreover, this insect species has a limited proportion of MUFA (<10% of total FA) and PUFA (<15% of total FA), with C18:1 *n*-9 (oleic) and C18:2 *n*-6 (linoleic) being the main FA for the two fractions, respectively [47]. Results about the FA profile of the laying quails’ diets further confirm this speculation. However, compared to the linear effect exerted by the FA composition of the defatted BSF larvae meal on the FA profile of quail and chicken meat [8,46], the FA profile of the eggs revealed that, even if the FA composition of the diets had a great impact, which confirms existing knowledge on this aspect [48], different mechanisms than the sole effect of the diet played a role.

To a great increase in the C12:0 content of the diets due to the inclusion of defatted BSF larva meal (+134%), the increase in the eggs did not show the same magnitude (+8%). On the other hand, to the relatively small dietary contents of palmitic acid (12.3% of total FA) and C18:0 (stearic acid; 5.67% of total FA), it was observed a double and more than 4-fold contents of C16:0 and C18:0 in the yolk, respectively. Results of the present research, in this sense, agreed with those by Secci et al. [11] on laying hens. Defatted BSF larva meal provided relevant amounts of C12:0, C14:0 and C16:0 SFA which were effectively desaturated and elongated by laying quails to C14:1 *n*-9 (myristoleic), C17:1 *n*-10 (Cis-10-Heptadecenoic), C16:1 *n*-9 (palmitoleic), C18:1 *n*-9 and C18:1 *n*-11 (vaccenic) acids, thus explaining the increasing trend in the MUFA content from Control to the BSF15 group.

The dietary content of linoleic acid and C18:3 *n*-3 (α-linolenic) acid in poultry diets is very important as they are essential precursors of longer *n*-6 and *n*-3 PUFA, though synthesis catalyzed by enzyme complexes, including Δ5 and Δ6-desaturases and elongases [49]. This was highlighted by the results of our study where laying quails produced eggs containing C20:4 *n*-6 (arachidonic), C20:5 *n*-3 (eicosapentaenoic; EPA) and C22:6 *n*-3 (docosaesaenoic; DHA) acids, even if diets had extremely low or no contents of such FA. In this sense, the capability of quails to enrich arachidonic acid and DHA contents in the egg yolk is documented [50]. As expected, the lower content of the essential precursors in the diet with increasing inclusion levels of defatted BSF larvae meal were the reason behind the lower content of the precursors themselves, but also of their derivatives, in the egg yolk.

Results of the present study agreed with other research where the dietary inclusion of feed ingredients with distinctive FA profile such as the hemp seed [51], flaxseed and stearidonic acid-enriched (C18:4 *n*-3) soybean oils [47], modified the FA composition of chickens’ egg yolk. The necessity to improve the FA profile of the larvae through substrate modulation in order to guarantee meat of satisfactory healthiness, was pointed out by previous papers on quails [8] and chickens [32,47], where an increase in the saturation degree of lipids was observed to the expenses of the unsaturated fractions. Differently, the intense change in the FA composition (% of total FAME) of quail eggs due to the dietary inclusion of defatted BSF larva meal, and the consequent changes also in FA contents (mg/100 g egg) were in contrast with the only other work testing the BSF larva meal as feed ingredient for layers diets [11], where the FA profile of chicken eggs was slightly affected by the dietary treatment. Such finding highlights thus the necessity of further research investigations to clarify the effect of this emerging feed ingredient on the FA profile of eggs in different poultry species. A dietary improvement of the FA composition of the diet seems however a desirable aspect to take into consideration in order to ensure heathy eggs for consumers.

The pH values recorded during the storage trial on quails’ eggs were slightly lower than values presented by Silva et al. [18]. This finding was not surprising as albumen pH gives a clear indication of the freshness of the egg and, in the present experiment, pH measurement was conducted the day after the eggs were laid. The observed increase in the albumen pH after 28 days of storage, which was independent to the dietary treatment, is the natural consequence of the loss of carbon dioxide during storage, and the breaking down of the ovomucin-lysozyme complex [52]. Literature data show that the supplementation of antioxidants-rich herbs or extracts from herbs during the laying period reduce eggs susceptibility to oxidative phenomena, thus offering the potential to increase the oxidative stability of poultry products [52]. The effect of a dietary supplementation with insect meal on the oxidative stability of the eggs has not been investigated yet, thus results of the present research are the first ones in this sense. Results of the present research agreed with those of Yesilbag et al. [53] where, despite the first day of storage MDA values didn’t differ among treatments, a dietary inclusion with rosemary and oregano oils lowered the MDA content of the quail eggs’ yolk during the refrigerated storage. Our finding in this sense could depend on two different aspects: As it was pointed out by other studies [8,40], the BSF larva contain carotenoids which are known for their antioxidant activity, which was however not assessed in the present study. On another hand, the defatted BSF larva meal is a source of SFA which determined a progressive lowering in the FA peroxidability index compared to the Control group, thus lowering the susceptibility to oxidative phenomena. Despite this, the observed lower MDA content in the BSF10 yolk, but not those of the BSF15 group compared to the Control yolks, was not expected and requires further investigation.

The sensory profile of eggs is surely a relevant aspect that must be considered when testing a novel feed ingredient in layers’ diets, as it demonstrated to be either negatively, positively or neutrally affected: Previous researches showed that the inclusion of fish silage [54] and flaxseed [55] in laying hens’ diets negatively affected some sensory traits of the eggs. Also, when turmeric was fed to laying quails some negative effects on yolk appearance were observed, but aroma and flavor were overall acceptable [18]. In literature the only other research study testing the effect of a dietary inclusion of defatted BSF larvae meal in layers’ diets on the sensory profile of the derived eggs, observed an improvement the appearance, texture, and taste of eggs derived from insects-fed birds (5% dietary inclusion), positively affecting the overall product acceptability [12]. Sensory improvements were attributable to the high glutamic acid content of the insect meal. The present defatted BSF larva meal was previously also tested in the diets for broiler quails at the same inclusion levels of the present study [8], but no differences in the sensory profile of the meat were observed. Analogously, in the present study the sensory traits of eggs derived from defatted BSF larva meal-fed quails were comparable with those obtained for quails fed the conventional diet. The latter result is extremely important in the perspective of a successful practical application of this new feed ingredient into dietary formulations for laying quails. 

## 5. Conclusions

Based on the results of the present experiment, the defatted black soldier fly larvae meal can be considered a possible alternative ingredient to soybean meal in laying quail diets, up to the 15% inclusion level. No negative effects on the productive performance of birds were observed and eggs showed overall satisfactory physicochemical traits and sensory profile. Future studies should deeply investigate the impact of this feed ingredient on eggs’ fatty acid profile as contrasting results are currently available on this aspect. Furthermore, an improvement in the fatty acid composition of the larvae or a dietary incorporation of a PUFA-rich ingredient, together with the insect seems desirable to ensure healthy food products in line with modern consumers’ exigencies.

## Figures and Tables

**Table 1:** Ingredients of the experimental diets (g/kg as fed) ^1^.

Ingredients	Experimental Diets		
	Control	BSF10 ^3^	BSF15 ^3^
Maize	483	524	546
Wheat	30.0	49.6	54.3
Soybean meal	359	230	166
Insect meal ^1^	0.00	100	150
Soybean oil	46.0	18.5	5.50
Calcium carbonate	70.5	68.0	68.0
Dicalcium phosphate	1.50	0.00	0.00
Salt	2.70	2.70	2.70
Methionine	2.40	2.40	2.40
Lysine	0.30	0.30	0.30
Vitamin-mineral premix ^2^	5.00	5.00	5.00

^1^ Defatted black soldier fly (BSF) larvae (*Hermetia illucens*). ^2^ Vitamin and mineral premix provided the following per kg of diet: Vitamin A, 11,500 IU; cholecalciferol, 2100 IU; vitamin E (from dl-tocopherylacetate), 22 IU; vitamin B12, 0.60 mg; riboflavin, 4.4 mg; nicotinamide, 40 mg; calcium pantothenate, 35 mg; menadione (from menadione dimethyl-pyrimidinol), 1.50 mg; folic acid, 0.80 mg; thiamine, 3 mg; pyridoxine, 10 mg; biotin, 1 mg; choline chloride, 560 mg; ethoxyquin, 125 mg; Mn (from MnSO_4_·H_2_O), 65 mg; Zn (from ZnO), 55 mg; Fe (from FeSO_4_·7H_2_O), 50 mg; Cu (from CuSO_4_·5H_2_O), 8 mg; I (from Ca (IO_3_)2·H_2_O), 1.8 mg; Se, 0.30 mg; Co (from Co_2_O_3_), 0.20 mg; Mo, 0.16 mg. ^3^ BSF10 and BSF15 are diets corresponding to 10% and 15% H inclusion levels, respectively.

**Table 2:** Chemical composition (g/kg as is) and gross energy (MJ/kg) content of the defatted BSF (black soldier fly) larvae meal.

Item	Amount
Dry matter	946
Crude protein	518
Ether extract	148
Ash	72.7
Calcium (Ca)	9.77
Phosphorus (P)	8.33
Ca/P	1.17
Gross energy	21.8

The methods to analyze the mineral content of larvae and experimental diets were: method 968.08 (Ca) and method 995.11 (P) of the AOAC [24]. Experimental diets were analyzed in triplicate for chitin content following the method by Zhang and Zhu [25] with the modifications described in Woods et al. [26].

**Table 3:** Chemical composition and gross energy content of the experimental diets (g/kg as is).

Measured Nutrients Composition	Experimental Diets		
	Control	BSF10	BSF15
Dry matter	906	908	909
Crude protein	194	200	195
Ether extract	69.6	52.8	53.7
Ash	113	107	105
Starch	287	319	347
Chitin	0.00	3.92	6.20
Calcium (Ca)	38.8	32.0	33.6
Phosphorous (P)	3.39	3.49	3.67
Ca/P	11.4	9.16	9.16
Gross energy (MJ/kg)	16.4	16.5	15.8

BSF10 and BSF15 are diets corresponding to 10% and 15% BSF larvae meal inclusion levels, respectively.

**Table 4:** Fatty acid profile (% of total fatty acid methyl esters (FAME)) of the experimental diets.

Fatty Acids	Experimental Diets		
	Control	BSF10	BSF15
C6:0	0.07	0.08	0.09
C10:0	0.00	0.03	0.47
C12:0	0.15	12.8	20.1
C14:0	0.11	2.63	4.11
C15:0	0.00	0.05	0.06
C16:0	11.5	12.3	13.03
C17:0	0.08	0.09	0.10
C18:0	2.51	2.38	2.33
C20:0	0.06	0.06	0.06
C22:0	0.13	0.10	0.08
C23:0	0.05	0.05	0.05
C24:0	0.00	0.04	0.04
Total SFA	14.6	30.9	40.6
C14:1 *n*-9	0.00	0.05	0.08
C16:1 *n*-9	0.10	0.69	1.13
C17:1 *n*-10	0.06	0.07	0.12
C18:1 *n*-9	24.4	20.8	18.8
C18:1 *n*-11	1.22	0.76	0.61
C20:1 *n*-9	0.36	0.32	0.30
C22:1 *n*-9	0.32	0.22	0.16
C24:1 *n*-9	0.66	0.44	0.15
Total MUFA	27.1	23.4	21.3
C18:2 *n*-6	51.6	40.6	33.6
C18:3 *n*-6	0.07	0.08	0.05
C20:2 *n*-6	0.05	0.08	0.06
C20:3 *n*-6	0.05	0.09	0.04
C20:4 *n*-6	0.05	0.06	0.05
C22:2 *n*-6	0.39	0.04	0.04
C18:3 *n*-3	4.39	2.90	2.12
C20:3 *n*-3	0.05	0.05	0.05
C20:3 *n*-3	0.08	0.08	0.07
C22:6 *n*-3	0.00	0.00	0.04
Total PUFA	56.7	43.8	36.1
Total *n*-6	52.2	40.8	33.9
Total *n*-3	4.52	3.03	2.28
*n*-6/*n*-3	11.5	13.5	14.8
Identified FA, %	98.4	98.2	98.0

SFA = Saturated fatty acids; MUFA = Monounsaturated fatty acids; PUFA = Polyunsaturated fatty acids. BSF10 and BSF15 are diets corresponding to 10% and 15% BSF larvae meal inclusion levels, respectively.

**Table 5:** Effect of the dietary inclusion of 0% (Control), 10% (BSF10) and 15% (BSF15) defatted BSF larvae meal on the productive performance of laying quails.

Items	Experimental Groups			Significance	RSD ^1^
	Control	BSF10	BSF15		
N. of laying quails	75	75	75		
Initial live weight, g	365	368	366	ns	5.31
Final live weight, g	352	361	356	ns	5.47
N. replicated cages	5	5	5		
Feed conversion ratio ^2^					
Week 1	3.31	3.29	3.23	ns	0.35
Week 2	3.98	3.43	3.46	ns	0.40
Week 3	3.92	3.62	3.71	ns	0.44
Week 4	3.71	3.86	3.71	ns	0.50
Week 5	3.09	2.86	2.95	ns	0.35
Weeks 1–5	3.60	3.40	3.40	ns	0.32
Total eggs, n.					
Week 1	70.2	68.6	66.8	ns	8.26
Week 2	78.2	78.0	80.0	ns	9.38
Week 3	78.6	76.6	73.0	ns	8.18
Week 4	79.8	75.2	76.6	ns	11.5
Week 5	85.0	82.8	82.2	ns	12.3
Weeks 1–5	392	381	379	ns	43.4
Egg production (%) ^3^					
Week 1	78.0	76.8	74.2	ns	9.32
Week 2	74.5	75.3	77.1	ns	0.35
Week 3	74.9	75.1	70.7	ns	7.96
Week 4	77.9	74.4	74.7	ns	9.91
Week 5	73.0	75.9	70.9	ns	8.63
Weeks 1–5	75.5	75.5	73.4	ns	7.41
Average egg weight (g)					
Week 1	14.2	14.5	14.3	ns	0.40
Week 2	14.2	14.6	14.3	ns	0.35
Week 3	14.4	14.7	14.6	ns	0.50
Week 4	14.4	14.6	14.5	ns	0.46
Week 5	14.3	14.7	14.5	ns	0.43
Weeks 1–5	14.3	14.6	14.4	ns	0.39
Defected eggs (%)^4^					
Week 1	2.28	1.41	2.14	ns	2.14
Week 2	1.34	2.33	3.06	ns	1.50
Week 3	2.32	3.02	2.24	ns	2.50
Week 4	0.55	3.05	3.23	ns	2.40
Week 5	2.08	0.65	2.75	ns	2.37
Weeks 1–5	1.71	2.10	2.74	ns	1.59
Mortality (%)					
Week 1	0.00	1.33	0.00	ns	1.72
Week 2	0.00	0.00	1.33	ns	1.72
Week 3	1.33	2.66	1.33	ns	3.22
Week 4	1.33	2.66	0.00	ns	3.85
Week 5	1.33	2.96	2.75	ns	3.64
Weeks 1–5	4.00	9.33	5.33	ns	5.84

^1^ RSD: Residual Standard Deviation. ^2^ Feed conversion ratio: Total feed intake/Total egg weight. ^3^ Egg production: (Number (n) of eggs produced/n laying quails) × 100. ^4^ Defected eggs: Eggs with defects (i.e., broken, without solid shell, with unusual shape).

**Table 6:** Effect of the dietary inclusion of 0% (Control), 10% (BSF10) and 15% (BSF15) defatted BSF larvae meal on eggs physical traits.

Physical Attributes	Experimental Groups			Significance	RSD ^1^
	Control	BSF10	BSF15		
N. eggs	210	210	210		
Egg weight (g)	14.3	14.6	14.5	ns	1.18
Egg shape index (%) ^2^	75.3 ^B^	76.3 ^A^	76.3 ^A^	<0.01	3.27
Surface area (cm^2^) ^3^	26.0	26.3	26.2	ns	1.52
Shell weight (g)	1.44 ^B^	1.51 ^A^	1.52 ^A^	<0.001	0.16
Shell thickness (mm)	0.214	0.210	0.213	<0.05	0.02
Shell percentage ^4^	10.1 ^B^	10.4 ^A^	10.5 ^A^	<0.001	1.02
Edible portion (%) ^5^	89.9	89.6	89.5	ns	1.12
Albumen pH	9.16	9.15	9.18	ns	0.23
Yolk colour ^6^	4.99 ^B^	5.57 ^Ab^	5.81 ^Aa^	<0.001	0.92

^1^ RSD: Residual Standard Deviation. ^2^ Egg shape index: (Diameter 1/Diameter 2) × 100. ^3^ Surface area: [(Egg weight)^0.7056^] × 3.9782. ^4^ Shell percentage: (Shell weight/egg weight) × 100. ^5^ Edible portion (g)/egg weight (g) × 100. ^6^ Roche yolk color fan (Vuilleumier, 1969). ^a–b^ Means in the same row with different superscript letters differ for *p* < 0.05. ^A–B^ Means in the same row with different superscript letters differ for *p* < 0.01.

**Table 7:** Effect of the dietary inclusion of 0% (Control), 10% (BSF10) and 15% (BSF15) defatted BSF larvae meal on eggs proximate composition (g/100 g) and cholesterol content.

Items	Experimental Groups			Significance	RSD ^1^
	Control	BSF10	BSF15		
N. samples	12	12	12		
Water	72.9	72.9	72.5	ns	0.50
Protein	12.2^a^	12.1 ^ab^	11.9 ^b^	<0.05	0.25
Lipids	12.6	12.8	13.1	ns	0.51
Ash	1.11^b^	1.19 ^ab^	1.29 ^a^	<0.05	0.15
Cholesterol (mg/100 g whole egg)	471	468	479	ns	22.5
Cholesterol (mg/whole egg)	68.2	67.9	69.5	ns	3.26

^1^ RSD: Residual Standard Deviation. ^a–b^ Means in the same row with different superscript letters differ for *p* < 0.05.

**Table 8:** Effect of the dietary inclusion of 0% (Control), 10% (BSF10) and 15% (BSF15) defatted BSF larvae meal on eggs fatty acids profile (% of total FAME).

Fatty Acids	Experimental Groups			Significance	RSD ^1^
	Control	BSF10	BSF15		
N. samples	12	12	12		
Total SFA	36.1 ^B^	37.8 ^A^	38.6 ^A^	<0.001	0.84
C12:0	0.03 ^Bb^	0.12 ^Ba^	0.25 ^A^	<0.001	0.08
C14:0	0.34 ^C^	1.08 ^B^	1.52 ^A^	<0.001	0.07
C15:0	0.05 ^AB^	0.05 ^B^	0.06 ^A^	<0.001	0.01
C16:0	24.1 ^B^	25.3 ^Ab^	26.0 ^Aa^	<0.001	0.50
C17:0	0.15 ^a^	0.10 ^b^	0.11 ^ab^	<0.05	0.04
C18:0	11.1 ^A^	10.8 ^AB^	10.2 ^B^	<0.01	0.62
C20:0	0.015 ^b^	0.020 ^ab^	0.030 ^a^	<0.05	0.01
C22:0	0.08 ^B^	0.10 ^B^	0.19 ^A^	<0.001	0.05
C24:0	0.23 ^A^	0.21 ^A^	0.15 ^B^	<0.001	0.04
Total MUFA	35.9 ^B^	38.6 ^A^	40.0 ^A^	<0.001	1.98
C14:1 *n*–9	0.05 ^C^	0.22 ^B^	0.36 ^A^	<0.001	0.03
C16:1 *n*–9	2.71 ^C^	3.87 ^B^	4.54 ^A^	<0.001	0.34
C17:1 *n*–10	0.11 ^B^	0.12 ^A^	0.13 ^A^	<0.001	0.01
C18:1 *n*–9	31.5	32.6	33.0	ns	1.73
C18:1 *n*–11	1.29 ^B^	1.46 ^Ab^	1.60 ^Aa^	<0.001	0.12
C20:1 *n*–9	0.12	0.13	0.14	ns	0.04
C22:1 *n*–9	0.04	0.04	0.04	ns	0.01
C24:1 *n*–9	0.12	0.22	0.19	ns	0.12
Total PUFA	25.5 ^A^	20.8 ^B^	18.7^C^	<0.001	1.40
C18:2 *n*-6	19.7 ^A^	15.4 ^Ba^	14.0 ^Bb^	<0.001	1.17
C18:3 *n*–6	0.22	0.21	0.22	ns	0.03
C20:2 *n*–6	0.04	0.04	0.04	ns	0.03
C20:3 *n*–6	0.11	0.13	1.10	ns	0.07
C20:4 *n*–6	3.50 ^a^	3.41 ^ab^	3.15 ^b^	<0.05	0.26
C18:3 *n*–3	0.61 ^A^	0.37 ^B^	0.26 ^B^	<0.001	0.14
C20:3 *n*–3	0.16 ^B^	0.18 ^AB^	0.20 ^A^	<0.05	0.04
C20:5 *n*–3	0.04	0.04	0.04	ns	0.01
C22:6 *n*–3	1.26 ^A^	1.06 ^B^	0.70 ^C^	<0.001	0.12
UFA:SFA	1.71 ^A^	1.57 ^B^	1.52 ^B^	<0.001	0.05
*n*–6	23.5 ^A^	19.2 ^Ba^	17.7 ^Bb^	<0.001	1.33
*n*–3	2.07 ^A^	1.65 ^B^	1.20 ^C^	<0.001	0.13
*n*–6/*n*–3	11.4 ^B^	11.7 ^B^	14.6 ^A^	<0.001	0.85
AI ^2^	0.41 ^C^	0.50 ^B^	0.55 ^A^	<0.001	0.02
TI ^3^	1.08 ^C^	1.18 ^B^	1.23 ^A^	<0.001	0.03
PI ^4^	46.9 ^A^	40.5 ^B^	35.1 ^C^	<0.001	2.38
HH ^5^	2.32 ^A^	1.99 ^B^	1.86 ^C^	<0.001	0.07

SFA = Saturated fatty acids; MUFA = Monounsaturated fatty acids; PUFA = Polyunsaturated fatty acids; UFA = Unsaturated fatty acids. ^1^ RSD: Residual Standard Deviation. ^2^ AI: Atherogenicity index = (C12:0 + 4 × C14:0 + C16:0)/[Total MUFA + Total (*n*-6) + total (*n*-3)]. ^3^ TI: Thrombogenicity index = (C14:0 + C16:0 + C18:0)/[(0.5 × total MUFA) + 0.5 × (*n*-6) + 3 x (*n*-3)/(*n*-6)]. ^4^ PI: Peroxidability index = (% monoenoic × 0.025) + (% dienoic × 1) + (% trienoic × 2) + (% tetraenoic × 4) + (% pentaenoic × 6) + (% hexaenoic × 8). ^5^ HH: Hypocholesterolemic/Hypercholesterolemic index = [(C18:1 *n*-9 + C18:2 *n*-6 + C20:4 *n*-6 + C18:3 *n*-3 + C20:5 *n*-3 + C22:5 *n*-3 + C22:6 *n*-3)/(C14:0 + C16:0). ^a–b^ Means in the same row with different superscript letters differ for *p* < 0.05. ^A–C^ Means in the same row with different superscript letters differ for *p* < 0.01.

**Table 9:** Effect of the dietary inclusion of 0% (Control), 10% (BSF10) and 15% (BSF15) defatted BSF larvae meal on the fatty acid profile (mg/100 g egg) of laying quail eggs.

Fatty Acids	Experimental Groups			Significance	RSD ^1^
	Control	BSF10	BSF15		
N. samples	12	12	12		
Total SFA	1127 ^b^	1207 ^ab^	1240 ^a^	<0.05	99.3
C12:0	0.77 ^Bb^	3.93 ^Ba^	7.87 ^A^	<0.001	2.47
C14:0	10.5 ^C^	34.5 ^B^	48.6 ^A^	<0.001	3.83
C15:0	1.67 ^AB^	1.54 ^B^	1.98 ^A^	<0.01	0.34
C16:0	752 ^b^	809 ^ab^	836 ^a^	<0.05	67.4
C17:0	4.59 ^a^	3.06 ^b^	3.50 ^ab^	<0.05	1.31
C18:0	347	345	329	ns	32.7
C20:0	0.47 ^b^	0.63 ^ab^	0.96 ^a^	<0.05	0.43
C22:0	2.52 ^B^	3.04 ^B^	6.17 ^A^	<0.001	1.69
C24:0	7.10 ^A^	6.77 ^A^	4.85 ^B^	<0.01	1.44
Total MUFA	1125 ^b^	1235 ^ab^	1288 ^a^	<0.05	126
C14:1 *n*–9	1.61 ^C^	7.14 ^B^	11.4 ^A^	<0.001	1.09
C16:1 *n*–9	84.5 ^C^	124 ^B^	146 ^A^	<0.001	16.0
C17:1 *n*–10	3.38 ^Bb^	3.96 ^ABa^	4.12 ^A^	<0.01	0.48
C18:1 *n*–9	986	1041	1063	ns	108
C18:1 *n*–11	40.5 ^Bb^	46.5 ^ABa^	51.4 ^A^	<0.001	5.35
C20:1 *n*–9	3.79	4.29	4.59	ns	1.19
C22:1 *n*–9	1.22	1.34	1.15	ns	0.41
C24:1 *n*–9	3.61	6.95	5.96	ns	3.64
Total PUFA	800 ^A^	664 ^Ba^	601 ^Bb^	<0.001	57.2
C18:2 *n*-6	615 ^A^	491 ^B^	450 ^B^	<0.001	46.4
C18:3 *n*–6	6.75	6.51	6.86	ns	0.87
C20:2 *n*–6	1.13	1.29	1.41	ns	0.81
C20:3 *n*–6	3.54	4.12	3.48	ns	2.20
C20:4 *n*–6	108	108	101	ns	9.91
C18:3 *n*–3	19.2 ^A^	11.5 ^B^	8.32 ^B^	<0.001	3.91
C20:3 *n*–3	4.86 ^b^	5.83 ^ab^	6.61 ^a^	<0.05	1.41
C20:5 *n*–3	1.11	1.40	1.21	ns	0.38
C22:6 *n*–3	39.5 ^A^	33.8 ^B^	22.3 ^C^	<0.001	4.34
*n*–6	735 ^A^	611 ^B^	563 ^B^	<0.001	54.7
*n*–3	64.6 ^A^	52.5 ^B^	38.5 ^C^	<0.001	4.17

^1^ RSD: Residual Standard Deviation. ^a–b^ Means in the same row with different superscript letters differ for *p* < 0.05. ^A–C^ Means in the same row with different superscript letters differ for *p* < 0.01.

**Table 10:** Effect of the dietary inclusion of 0% (Control), 10% (BSF10) and 15% (BSF15) defatted BSF larvae meal on eggs oxidative status (TBARS, expressed as mg MDA/kg egg yolk) and albumen pH.

Item	Experimental Groups			Significance-Diet	RSD ^1^
	Control	BSF10	BSF15		
N. samples	16	16	16		
pH:					
Day 0	8.64	8.77	8.73	ns	0.20
Day 28	9.41	9.42	9.50	ns	0.12
Significance-day	<0.001	<0.001	<0.001		
RSD	0.21	0.17	0.09		
TBARs:					
Day 0	1.72	1.82	1.78	ns	0.08
Day 28	1.72 ^A^	1.60 ^B^	1.66 ^AB^	<0.01	0.06
Significance-day	ns	<0.001	ns		
RSD	0.04	0.05	0.11		

MDA = malondialdehyde; TBARs = thiobarbituric acid-reactive substances. ^1^ RSD: Residual Standard Deviation; ^a–c^ Means in the same row with different superscript letters differ for *p* < 0.05. ^A–B^ Means in the same row with different superscript letters differ for *p* < 0.01.

**Table 11:** Effect of the dietary inclusion of 0% (Control), 10% (BSF10) and 15% (BSF15) defatted BSF larvae meal on the sensory scores of quail eggs.

Sensory Attributes	Experimental Groups			Significance	RSD ^1^
	Control	BSF10	BSF15		
N. samples	60	60	60		
Yolk:					
Odor intensity	5.87	5.80	5.87	ns	0.38
Aroma intensity	6.09	6.10	6.04	ns	0.42
Taste:					
Sweetness	3.54	3.44	3.43	ns	0.58
Saltiness	3.69	3.53	3.51	ns	0.51
Texture:					
Solubility	5.01	5.10	5.05	ns	0.73
Adhesiveness	6.19	6.02	6.04	ns	0.58
Albumen:					
Odor intensity	5.38	5.37	5.39	ns	0.44
Aroma intensity	5.30	5.35	5.33	ns	0.37
Taste:					
Saltiness	3.00	2.91	2.96	ns	0.51
Texture:					
Consistency	5.69	5.63	5.54	ns	0.73

^1^ RSD: Residual Standard Deviation.

**Table 12:** Effect of the dietary inclusion of 0% (Control), 10% (BSF10) and 15% (BSF15) defatted BSF larvae meal on yolk and albumen off-flavors perception (% on total tested samples).

Sensory Attributes	Experimental Groups			Significance	χ^2^
	Control	BSF10	BSF15		
N. samples	60	60	60		
Yolk:					
Bitterness	2.78	2.78	8.33	ns	2.11
Sourness	0.00	2.78	0.00	ns	4.04
Metallic	0.00	2.78	5..55	ns	2.88
Poultry	16.7	11.1	8.33	ns	1.97
Grass	5.55	0.00	5.55	ns	3.10
Feed	5.55^b^	13.9^ab^	19.4^a^	<0.05	6.08
Fish	2.78	8.33	2.78	ns	2.11
Rancid	2.78	0.00	0.00	ns	4.04
Albumen:					
Bitterness	0.00	2.78	0.00	ns	4.04
Metallic	13.9	13.9	11.1	ns	0.10
Poultry	2.78	5.55	2.78	ns	0.30
Feed	2.78	8.33	0.00	ns	5.65

^a-b^ Means in the same row with different superscript letters differ for *p* < 0.05.
